# Visual-Tactile Phobic Hallucinations in a Child With Stimulant-Managed Attention-Deficit/Hyperactivity Disorder (ADHD)

**DOI:** 10.7759/cureus.20012

**Published:** 2021-11-29

**Authors:** Martin Leczycki, Eric MacMaster

**Affiliations:** 1 Department of Psychiatry, State University of New York (SUNY) Upstate Medical University, Syracuse, USA

**Keywords:** pediatric, stimulant, adhd, hallucinations, phobic, tactile, visual, vtph

## Abstract

Hallucinations are not uncommon in the pediatric population, and they can occur in a variety of presentations influenced by medical and non-medical factors. This case report summarizes existing literature concerning visual-tactile phobic hallucinations (VTPH) and describes a case with a unique presentation: VTPH in a child with stimulant-managed attention-deficit/hyperactivity disorder (ADHD). Observations made in this case and in those like it are used to characterize VTPH as a distinct psychiatric phenomenon that is observed in children without metabolic, neurologic, or other medical involvement. When it is a primary psychiatric symptom, VTPH typically has an acute but benign course that benefits not from a medically intensive approach, but rather one that explores psychosocial influences and provides reassurance and support.

## Introduction

Hallucinations are false sensory perceptions not associated with any identifiable external stimuli. In children, they should be differentiated from similar age-appropriate phenomena such as eidetic images, imaginary companions, and other fantasies and illusions [1]. Hallucinations have been culturally perceived as indicators of psychopathology, but in children, they can be a part of normal development [2]. Pathological causes of hallucinations can fall into the realm of metabolic, neurologic, psychiatric, or other medical involvement. It is important for physicians to recognize and treat these causes promptly as they may be life-threatening. Other causes of hallucinations include psychosocial adversity and other nonpsychotic psychopathology such as anxiety or phobia [1,2]. These known, but less frequently reported causes should remain on the differential diagnosis of a pediatric patient with hallucinations. While it is known that certain medications, including stimulants and other medications with dopaminergic effects, can induce symptoms of psychosis [3-5], ultimately the cause of many hallucinations cannot be confidently explained. The following case describes the differential diagnosis and management of a pediatric patient with acute-onset visual-tactile phobic hallucinations (VTPH) who had been taking extended-release dextroamphetamine for attention-deficit/hyperactivity disorder (ADHD), a unique presentation that has not been reported in the literature.

## Case presentation

The patient was a six-year, 10-month-old male with a past medical history of ADHD diagnosed at five years old who had benefited from extended-release dextroamphetamine prescribed by his pediatrician. Over time, the dose was titrated up to 20 mg daily for the last month prior to evaluation. Side effects had been limited to intermittent abdominal discomfort. He did not take any other medications, nor did he have other significant medical or psychiatric history. There were no complications during pregnancy, delivery, or early childhood. The family composition included his biological parents and two siblings. Family history was significant for anxiety in several family members, a paternal grandfather with schizophrenia, and a younger sister with seizures. No abuse or trauma histories were reported by either the patient or parents. He was seen by his pediatrician within the week prior to presenting to the hospital; collateral information from the pediatrician indicated he was in his normal state of health and his ADHD was well-managed. There was no indication that medications were being mishandled; parents reported that given the age of children in the home, medications were kept well out of reach. Nobody in the home was taking herbal supplements, that if taken by the patient, could have interacted with his dextroamphetamine.

On the night of the episode, the patient awoke at three in the morning, then began to describe visual and tactile hallucinations, specifically seeing and feeling spiders in his room, on his body, and in his mouth. He made comments such as “I feel like spiders are crawling on me” and “I feel like I am on fire.” His parents confirmed no spiders were present and attempted to console him, but he became increasingly fearful and agitated. On the way to the Emergency Department, he was screaming without fluent speech, flailing his arms, gesturing to his mouth, and making motions as though to remove bugs from his body. His parents reported he had a preexisting minor insect phobia but was usually easily consoled, and that he had nightmares and night terrors previously but never anything like this episode. His parents felt confident that he was awake and conscious during the episode.

While in the Emergency Department, the patient was persistently agitated, crying inconsolably, and continued to report visual and tactile hallucinations. Though a complete physical and neurological exam was not possible at the time, he was afebrile but tachycardic, tachypneic, hypertensive, and responding to internal stimuli. He received lorazepam 0.5 mg IV several times with little improvement in agitation. Upon transfer to the Pediatric Intensive Care Unit, he was started on dexmedetomidine 0.5 mg/kg/hr IV for sedation that was continued on the first day. At this time, his dextroamphetamine was stopped. For the next two days, he continued to report seeing spiders in his room at times, causing distress. However, these episodes eventually lessened in frequency and severity, and he was once again easily consoled by his parents. After a three-day hospital stay, he was discharged home.

On the day after admission, a physical exam revealed a well-appearing, normal stature boy in no acute distress. He had normal vital signs. He had a single café au lait macule over the right nipple. The neurological exam was grossly intact but notable for mild postural tremor of the bilateral upper extremities. Mental status exam revealed a fidgety, anxious, and hypervigilant child worried about his hallucinations returning. He appeared, spoke, and behaved appropriately for his age. As shown in Table 1, medical workup was largely negative or within normal limits; it consisted of CMP, CBC with differential, ESR, CRP, TSH, T4, ammonia, folate, ceruloplasmin, cortisol, Lyme titers, UA, body fluid culture, COVID-19 PCR, CSF studies, and brain MRI. Urine toxicology was positive for amphetamines, which was expected due to the patient’s prescribed dextroamphetamine. The respiratory pathogen panel was positive for parainfluenza virus type 3. ECG performed on admission showed sinus tachycardia. EEG was not completed in the hospital; the patient would not tolerate it.

**Table 1:** Laboratory test results of the patient AST - aspartate aminotransferase; ALT - alanine aminotransferase; WBC - white blood cells; RBC - red blood cells; ESR - erythrocyte sedimentation rate; CRP - C-reactive protein; TSH - thyroid-stimulating hormone; CSF - cerebrospinal fluid

Test Name	Result	Reference Range
Albumin	4.0 g/dL	3.8-5.4 g/dL
Bilirubin	0.3 mg/dL	<1.2 mg/dL
Calcium	9.4 md/dL	8.8-10.8 md/dL
Chloride	105 nmol/L	98-107 nmol/L
Creatinine	0.48 mg/dL	0.32-0.59 mg/dL
Glucose	96 mg/dL	70-140 mg/dL
Alkaline Phosphatase	271 U/L	142-335 U/L
Potassium	4.2 nmol/L	3.4-5.1 nmol/L
Total Protein	6.7 g/dL	5.6-7.5 g/dL
Sodium	138 nmol/L	136-145 nmol/L
AST/SGOT	32 U/L	<40 U/L
Blood Urea Nitrogen (BUN)	7 mg/dL	5-18 mg/dL
Blood Osmolality	283 mosm/kg	275-300 mosm/kg
BUN/Cr Ratio	15	10-20
Bicarbonate	24 mmol/L	22-29 mmol/L
ALT/SGP	14 U/L	<41 U/L
Anion Gap	8 mL/min	8-15 mL/min
WBC	5.6 x 10^3/µL	4.5-13.0 x 10^3/µL
RBC	4.3 x 10^6/µL	4.0-5.2 x 10^6/µL
Hemoglobin	11.5 g/dL	11.5-15.5 g/dL
Hematocrit	34.6%	35.0-45.0%
Mean Cell Volume	80.5 fL	77.0-96.0 fL
Mean Cell Hemoglobin	26.7 pg	25.0-31.0 pg
Mean Cell Hemoglobin Conc	33.2 g/dL	32.0-36.0 g/dL
Red Cell Dist Width	13.7%	11.5-14.5%
Platelet Count	216 x 10^3/µL	150-400 x 10^3/µL
Neutrophil Differential	42%	45-75%
Lymphocyte Differential	42%	19-46%
Monocyte Differential	12%	2-12%
Eosinophil Differential	3%	0-4%
Basophil Differential	1%	0-1.5%
Abs Neutrophil	2.34 x 10^3/µL	1.50-8.00 x 10^3/µL
Abs Lymphocyte	2.39 x 10^3/µL	1.50-7.00 x 10^3/µL
Abs Monocyte	0.65 x 10^3/µL	0.00-0.80 x 10^3/µL
Abs Eosinophil	0.17 x 10^3/µL	0.00-0.50 x 10^3/µL
Abs Basophil	0.04 x 10^3/µL	0.00-0.20 x 10^3/µL
ESR	4.0 mm/hr	<15 mm/hr
CRP	<3.0 mg/L	<8.0 mg/L
TSH	1.62 IU/mL	0.60-4.80 IU/mL
T4	5.4 µg/dL	6.0-13.8 µg/dL
Ammonia	18 µmol/L	16-60 µmol/L
Folate	16.30 ng/mL	>4.77 ng/mL
Ceruloplasmin	23 md/dL	15-30 md/dL
Cortisol	12.2 µg/dL	-
Lyme Titer	IgG negative; IgM negative	-
Urine Color	Light yellow	-
Urine Appearance	Clear	-
Urine Specific Gravity	1.017	1.010-1.025
Urine pH	6.5	4.5-8.0
Urine Protein	Negative	-
Urine Glucose	Negative	-
Urine Ketone	Negative	-
Urine Bilirubin	Negative	-
Urine Blood	Negative	-
Urine Leukocyte Esterase	Negative	-
Urine Nitrite	Negative	-
Urine Toxicology Panel	Positive for amphetamines	-
Body Fluid Culture and Gram Stain	No WBCs or organisms detected	-
CSF Pathogen Panel	No pathogens detected	-
CSF Protein	33 mg/dL	<50 mg/dL
CSF Glucose	71 mg/dL	60-80 mg/dL
CSF Color	Colorless	-
CSF Clarity	Clear cells	-
CSF RBC Count	<2/µL	<2/µL
CSF Total Nucleated Cell Count	<3/µL	<7/µL
COVID PCR	2019-nCov not detected	-
Respiratory Pathogen Panel	PCR positive for parainfluenza virus type 3	-
ECG 12-Lead	Rate 136 bpm, QRS 64 ms, QTC 441 ms	-
EEG 24-hr Ambulatory	No epileptiform activity detected	-
MRI Brain	Normal	-

Aside from sedation with lorazepam and dexmedetomidine, the patient did not receive medications while hospitalized. His dextroamphetamine was stopped. The patient and his parents were provided with reassurance and were taught coping strategies, deep breathing exercises, and other interventional measures should the hallucinations return. The patient was also scheduled for therapy to be started soon after discharge from the hospital. One month later, the patient restarted his dextroamphetamine as prescribed by his pediatrician. At that time, 24-hour ambulatory EEG did not record any epileptiform activity. Two months after restarting dextroamphetamine, the patient’s hallucinations had not recurred.

## Discussion

For the patient described, a differential diagnosis was created. Various medical disorders can cause hallucinations and should be the first to be excluded. These include, but are not limited to, electrolyte imbalance, nutritional deficits, thyroid disease, Wilson’s disease, porphyria, adrenal disease, migraines, seizures, and infections such as meningitis and encephalitis [6]. Despite the patient testing positive for parainfluenza virus type three, post-infectious encephalitis was ruled out as the patient was afebrile, experienced no symptoms of infection other than abdominal pain on the day before the hallucinations began, lacked neurological symptoms which would be expected in encephalitis (e.g., headache, neck stiffness, change in alertness or cognition), and his brain MRI was normal. Occipital lobe epilepsy was supported by the occurrence of visual hallucinations and a family history of seizures in his sister. However, hallucinations are rarely caused by epilepsy and are typically brief, lasting just a few seconds [6]. In this patient, the initial hallucinatory episode lasted for several hours and intermittently recurred over the next few days. In addition, a 24-hour ambulatory EEG performed after hospital discharge did not detect any epileptiform activity. All other medical causes for the hallucinations were excluded due to negative exam and/or test results. Psychiatric causes were then considered.

It is unlikely that this acute episode of psychosis was caused by very early-onset schizophrenia, which is characterized by hallucinations, paranoia, delusions, and cognitive impairment in children under 13 years of age. This condition is very rare; its prevalence is estimated to be one in 30,000 children [7]. If this were the diagnosis, there would have likely been a premorbid period characterized by a recent decline in school performance, cognition, or change in affect, mood, or behavior preceding the psychotic symptoms, which likely would have persisted and worsened without treatment.

At first impression, stimulant-induced psychosis was high on the team’s differential. In fact, the usage of stimulant medications, which have known dopaminergic effects, is associated with hallucinations, delusions, and other psychotic phenomena in a small number of young children with ADHD. These symptoms usually resolve within two days after discontinuing the stimulant and almost always within one week [3]. Case narratives describe at least a dozen children taking dextroamphetamines for ADHD, with no concurrent medical conditions or other medications, presenting with hallucinations like those described in this case report. These hallucinations are commonly described as animals or insects [8], as with this patient. A review of insurance claims suggests that there may be an association between psychosis and stimulant use in adolescents, with acute-onset psychotic episodes occurring in one in 660 patients who were prescribed a stimulant for ADHD. These episodes occurred a median of 128 days after starting the medication and were more common in patients who were prescribed amphetamines rather than methylphenidate, and in patients receiving higher doses [4,9]. Similarly, pediatric clinical trials report few cases of new-onset psychosis in children treated for ADHD, but no such cases with placebo treatment [5]. In the case of this patient, he had been taking dextroamphetamine for 20 months with no adverse effects or hallucinations until this episode and did not report any hallucinations later after restarting dextroamphetamine. It is unlikely that his hallucinations were suddenly caused by his medication at this point, so alternative explanations were considered.

Children of similar age and presentation to this case have also been described as having VTPH, an uncommon phenomenon unique to this age group. A few reports describe children of early school age experiencing an acute episode of hallucinations at night. These children awoke unexpectedly, screaming and crying that they were seeing and feeling a phobia of theirs, most often insects, snakes, or rodents that were on or near their bodies. These fears were unable to be alleviated with reassurance. Unlike night terrors, which are common in this age group, these episodes were not associated with hypnagogic hallucinations. The children were alert and had a vivid memory of the events. The hallucinations were self-limited, occurring intermittently for the next few days and a subacute anxious-phobic period lasting up to two weeks. Once medical causes, medication reactions, overdoses, and toxin ingestions were ruled out and there was no evidence of abuse or Munchausen syndrome by proxy, these children were deemed to have VTPH as a diagnosis of exclusion [10-12]. It should also be noted that none of these children had been taking stimulants or psychiatric medications of any kind, making VTPH a separate diagnosis from stimulant-induced psychosis. Given the near exact description of this case (e.g., an early school-age child with acute onset of visual and tactile hallucinations of a phobia at night, unable to be consoled, lessening the severity, and frequency in the next few days), the rarity of psychosis in young patients who are prescribed stimulants for ADHD, and the inability to prove causality from stimulant use in this case, VTPH stands as a better explanation.

Studies of large pediatric samples show that hallucinatory phenomena may occur in 8%-21% of 11-year-old children [13,14], with most hallucinations being transient and resolving spontaneously. Many of these children lack a DSM diagnosis or significant medical history [2]. As such, these hallucinations may not be a sign of psychopathology or some medical disorder, but rather a form of poor reality testing affected by developmental issues, stress, deprivation, family relationships, or sociocultural factors [1,2]. VTPH may fit into this understanding of hallucinations, given that in the cases where it has occurred, the children were otherwise healthy. As nearly all reported cases of VTPH occur at night, there is also a possibility that the content of nightmares or hypnopompic hallucinations can slip into the conscious state upon awakening, where they can meet with existing fears or phobias, amplifying them to the point of uncontrollable fear, panic, and vivid hallucinatory phenomena. It is interesting to note that in this case, the patient was already known to have a minor phobia of insects, and his visual and tactile hallucinations were focused on this phobia.

While it is important to rule out common medical causes, Child Psychiatry should be involved early on to perform a thorough evaluation of the patient, perhaps examining the psychodynamic content of the hallucination or providing appropriate emotional and cognitive support. Interventional use of CBT has shown to be helpful as a form of reassurance to the patient but also as a means of exploring how the patient understands and explains their hallucinations, how they started, and whether they can be controlled or stopped [6]. Such an approach may prove to be helpful to patients with VTPH, as there is an anxious and/or phobic component that can likely be treated.

Regardless of the cause of any type of acute-onset hallucination in children, the majority of relevant cases in the literature describe a situation wherein by the time all medical tests and investigations have been completed in the following days, the hallucinations have subsided either completely or to a subacute level at which they can be easily managed with reassurance. This observation holds true for cases describing patients taking stimulants for ADHD [4,8,9] as well as patients with no significant medical or psychiatric history [10-12]. For this reason, it may be recommended to perform the most pertinent medical studies (e.g., serology, cultures, toxicology panels, ECG) while holding off on more costly or invasive studies (e.g., CT, MRI, LP), which are unlikely to change the management of the patient and may cause undue stress for the patient and their family, compounding with any other issues possibly influencing the hallucination. In any case, it would make sense to exercise a sensitive, trauma-informed approach to minimize the level of stress in the hospital and to provide reassurance.

## Conclusions

This case adds to the growing list of evidence for VTPH being a distinct psychiatric phenomenon, and as such, medical providers should be able to recognize the signs and symptoms of VTPH. The typical patient is a child of early school age, presenting at night with acute-onset visual, tactile, phobic hallucinations, and lacks evidence of metabolic, neurologic, or other medical involvement. As the more common causes of hallucinations are ruled out, Child Psychiatry should perform a thorough evaluation of the patient, look into psychosocial factors and environmental stressors that can be associated with VTPH, and provide appropriate reassurance and support. Regardless of the cause of the hallucinations described in this case, extensive medical workup has not been shown to affect the management of similar reported cases, which usually resolve spontaneously in a matter of days. It should also be noted that stimulant medications can induce psychotic symptoms in children, though reported cases are rare, and causality may be difficult to prove. VTPH has only yet been described in patients who were not taking stimulants, so while it could certainly be influenced by stimulant use, VTPH is a separate diagnosis from stimulant-induced psychosis.
